# Scheduled Support Versus Support on Demand in Internet-Delivered Cognitive Behavioral Therapy for Social Anxiety Disorder: Randomized Controlled Trial

**DOI:** 10.32872/cpe.11379

**Published:** 2023-09-29

**Authors:** Anton Käll, Cecilia Olsson Lynch, Kajsa Sundling, Tomas Furmark, Per Carlbring, Gerhard Andersson

**Affiliations:** 1 Department of Behavioural Sciences and Learning, Linköping University, Linköping, Sweden; 2 Department of Biomedical and Clinical Sciences, Linköping University, Linköping, Sweden; 3 Department of Psychology, Uppsala University, Uppsala, Sweden; 4 Department of Psychology, Stockholm University, Stockholm, Sweden; 5 Department of Clinical Neuroscience, Karolinska Institutet, Stockholm, Sweden; Philipps-University of Marburg, Marburg, Germany

**Keywords:** social anxiety disorder, ICBT, Internet-delivered treatments, guided ICBT

## Abstract

**Objectives:**

Clinician-supported internet-delivered cognitive behavioral therapy (ICBT) can be an effective treatment option when treating social anxiety disorder (SAD). Unguided ICBT is often found to be less effective. One possible solution to reduce the costs of clinician support is to provide support on demand. In this format of guidance, participants have the option to contact their clinician if needed. In a few studies, this mode of support has been compared favorably to scheduled support.

**Method:**

Participants in a previously reported controlled trial on SAD who had been in a waitlist control group were randomly allocated to ICBT with either on-demand guidance or scheduled weekly therapist guidance. A total of 99 participants were included. Data were collected weekly on the primary outcome measure, the Liebowitz Social Anxiety Scale self-report (LSAS-SR), and at pre- and post-treatment for secondary measures. Data were analyzed in accordance with the intention-to-treat principle using mixed-effects models.

**Results:**

Both groups improved significantly during the treatment according to the LSAS-SR ratings. The groups did not differ in their estimated change during the treatment period, with a between-group effect of d = 0.02, 95% CI [-0.37, 0.43]. Both groups experienced similar improvement also on the secondary outcome measures, with small between-group effect sizes on all outcomes.

**Conclusions:**

The findings indicate that support on demand can be an effective way of providing guidance in ICBT for SAD, although more research on this topic is needed. A limitation of the study is that it was conducted in 2009, and the findings were in the file drawer. Subsequent published studies support our initial findings, but more research is needed.

## Highlights

- One way to handle the need for therapist support in internet-delivered cognitive behaviour therapy (ICBT) is to offer support on demand.
- A randomized controlled trial was conducted comparing scheduled versus support on demand when completing ICBT for social anxiety disorder (SAD).
- Both groups improved and there were no major differences in outcome.
- Support on demand can be an effective way of providing guidance in ICBT for SAD.

Social anxiety disorder (SAD) is a common and debilitating mental health problem characterized by a persistent and intense fear of being evaluated in social situations (American Psychiatric Association, 2013). Global estimates suggest that SAD has an average lifetime prevalence of around 4%, often coupled with an early onset (Stein et al., 2017) and, when left untreated, a chronic course (Steinert et al., 2013).

Psychological treatments have been shown to assist people with this problem (Acarturk et al., 2009). Cognitive behavioral therapy (CBT) is often seen as the gold standard among these treatments, producing large effect sizes (Mayo-Wilson et al., 2014) and lasting effects that are maintained years after therapy termination (van Dis et al., 2020). Additionally, CBT targeting SAD has been disseminated successfully using modes other than traditional individual therapy, for example, in group settings (Barkowski et al., 2016) and via the Internet (Guo et al., 2021), most commonly in the form of internet-delivered CBT (ICBT; Andersson, 2018). ICBT provides a resource-effective way of delivering psychological treatment, as it requires less time from the therapist and can increase access to CBT in underserved areas and populations (Andersson, 2016). It has also been shown to be a cost-effective option (Donker et al., 2015). Specifically for SAD, ICBT has been shown to be an effective option in a regular care setting (El Alaoui et al., 2015) and to have lasting effects five years after termination (Hedman et al., 2011).

ICBT is often administered with scheduled support from a therapist (Andersson, 2016), and studies suggest that this is more effective than pure self-help versions of ICBT (Baumeister et al., 2014). However, there are exceptions. For example, one study conducted in China reported that a pure self-help condition produced comparable results to a condition which received regular therapist guidance (Kishimoto et al., 2016). Furmark et al. (2009) also found that a bibliotherapy condition with minimal therapist contact led to similar improvement compared to a therapist-supported ICBT condition, and that both active conditions outperformed a waitlist control group (Furmark et al., 2009).

One alternative to providing scheduled clinical support in ICBT is to provide support on demand (also referred to as optional support). This requires clients to contact their clinician when they want feedback, support, or have questions regarding the treatment material. This resembles helplines and usually requires less clinician time. Support on demand has been found to generate similar results to guided ICBT interventions in the treatment of anxiety and depression in a routine care setting (Hadjistavropoulos et al., 2017; Hadjistavropoulos et al., 2019). Additionally, the results indicated no significant differences in satisfaction with the treatment. Dear et al. (2015) did not find any significant differences between the optional support condition and scheduled therapist support in a trial on chronic pain, with high satisfaction and completion ratings across conditions (Dear et al., 2015). Support on demand has also been shown to have similar long-term outcomes compared to scheduled guidance in a study examining the outcomes of ICBT for loneliness two years after treatment (Käll et al., 2020). In a small factorial design trial on generalized anxiety disorder, the authors reported that support on demand was as effective as scheduled support, but that scheduled support was rated as more positive (Dahlin et al., 2022). Also, it has been suggested that scheduled guidance compared to optional guidance, is slightly more favorable at least in terms of adherence (Koelen et al., 2022). In conclusion, controlled trials on clinician support on demand provides initial support for this guidance format. This way of disseminating ICBT could increase access to ICBT and reduce costs for the support function while still not sacrificing effects and safety.

Given the increasing interest in ICBT and the need to make ICBT scalable, the aim of the current study was to compare the support on demand mode with scheduled support in ICBT treatment for SAD. Here we report findings from an unpublished part, i.e. a waiting list control group, of a previous randomized controlled trial (Andersson et al., 2012). After initial waiting-time individuals were randomized to the two forms of guidance. We had originally hypothesized that the support on demand group would experience smaller reductions in symptoms of social anxiety and related psychopathology and smaller increase in quality of life. In addition, the support on demand group was expected to lead to less demand for therapist input.

## Method

The current study was part of the SOFIE-6 project, a study investigating the efficacy of ICBT for SAD (Andersson et al., 2012). Here, we report the results from the waitlist control group, which received treatment directly following the first group in the controlled trial.

### Participants and Recruitment

**Figure 1:**
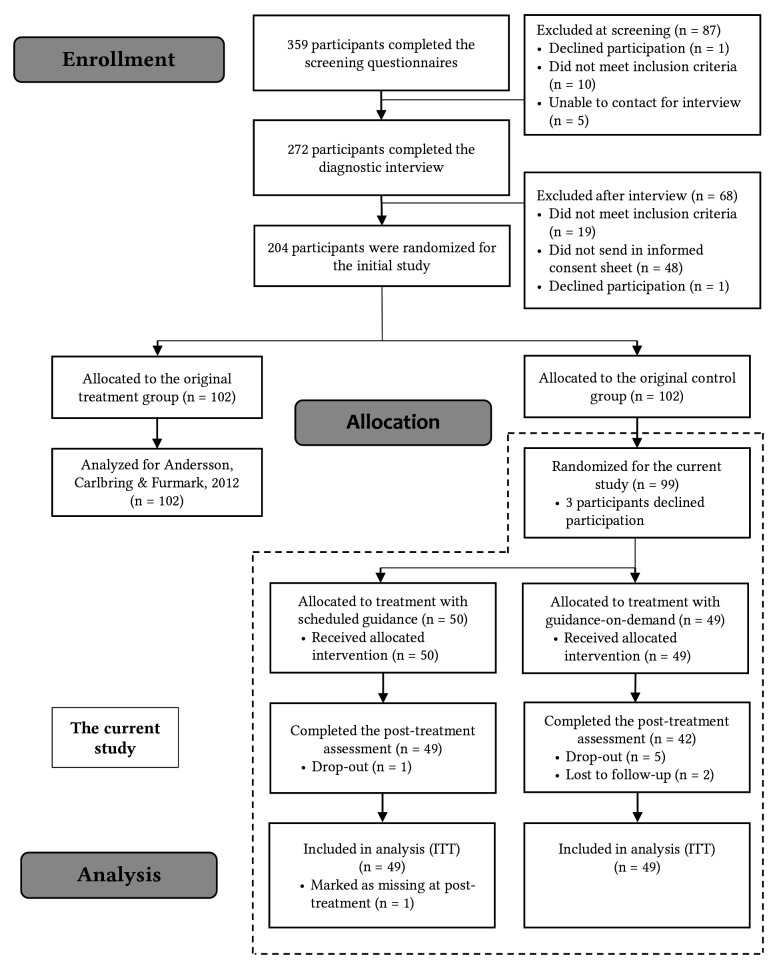
Flowchart of the Recruitment and Assessments Throughout the SOFIE-6 Study

A flowchart of the recruitment and treatment processes is presented in Figure 1. More information about the initial phase of the study can be found in Andersson et al. (2012). Participants were recruited via an email sent out to a waitlist who had registered interest on a public site hosted by the research group (www.studie.nu). An email invitation was sent to the first 600 names on the list. A total of 359 participants completed the screening questionnaires, and 272 completed the subsequent Structured Clinical Interview for DSM-IV Axis I Disorders (SCID-I) (First et al., 1997) via telephone. The SCID-I interviews were conducted by 10 final-year students from the clinical psychologist program at Uppsala University, Sweden. They received training in administering the interviews before the study. Inclusion criteria were: a) at least 18 years old, b) living in Sweden, c) having access to a computer and an internet connection, d) meeting the criteria for SAD on the Social Phobia Screening Questionnaire (SPSQ) (Furmark et al., 1999), e) meeting the SCID-I criteria for SAD without meeting the criteria for a comorbid eating disorder or psychotic disorder, f) if applicable, having a stable dose of medication for the past two months, g) not undergoing current psychological treatment or having received psychological treatment during the past six months, h) providing informed consent via mail. In the original study, 204 participants met these criteria and were randomized to receive treatment either immediately during the autumn of 2008 or later. Once the post treatment data were collected, the control group participants (*n* = 99 after accounting for dropout) were randomized once again using a true random number generator (www.random.org) to receive either scheduled support or support on demand. Randomization during both phases was conducted by researchers not involved in other aspects of the study.

### Treatment

The treatment was divided into nine modules that were unlocked one at a time, given that the participants had completed the assignments in the previous module. Modules were unlocked on a weekly basis, and participants were informed that this was the expected pace to keep during the treatment period. All unlocked modules were available for the duration of the treatment phase. Each module consisted of a PDF containing texts and practical exercises to complete during the week. A quiz was placed at the end of the modules boost adherence to the important principles of the treatment. Participants also provided a short written summary of the module in their weekly correspondence with the therapist (for the regular support group) or in a separate email to a non-specific therapist (in the support on-demand group). The content of the modules was identical to those used in previous studies within the SOFIE project (Furmark et al., 2009), which contained psychoeducation, cognitive restructuring, behavioral experiments, exposure exercises, and social skills training. An outline of the treatment is presented in Table 1. The modules spanned 188 pages, ranging from 17 to 30 pages per module.

**Table 1:** Content of the Modules

Module	Content	Exercises	Number of pages (A4)
**1**	Introduction and psychoeducation	Learning about symptoms, anxiety hierarchy	18
**2**	Clarks and Wells’ cognitive model of social anxiety	Personal model of social anxiety, thought record	20
**3**	Cognitive restructuring I	Reality testing, cognitive distortions, goals for the treatment	30
**4**	Cognitive restructuring II	Negative automatic thoughts, behavioral experiments	23
**5**	Exposure I	Exposure based on anxiety hierarchy	21
**6**	Shifting focus	Safety behaviors, exposure	19
**7**	Exposure II	Safety behaviors, exposure	17
**8**	Social skills	Social skills, exposure	19
**9**	Relapse prevention	Summary, plan for relapse prevention	21

Ten clinical psychologists served as clinicians during treatment. Communication between the participants and the clinician was conducted via a messaging system on the encrypted study website (Vlaescu et al., 2016). In addition to the messaging system, all participants had access to one of two anonymous discussion forums where they could write about the progress and experiences of the exercises conducted during the week. The scheduled support and support on demand groups had separate forums, and both forums were monitored by the study staff for safety. All participants received an introductory message, but for the group with scheduled support, this message was sent from their personal clinician, while the support on demand group participants received a generic message. The group with scheduled support received feedback on their exercises on a fixed day each week, which was the same day as when they received access to the next module. The support on demand group participants were told that they could contact the study staff via the messaging system. Participants in this condition were not assigned a specific clinician; rather, the clinicians had a schedule with days during which they would monitor the activity of the participants and respond to requests for help and feedback.

### Measures

#### Primary Outcome Measure

##### Liebowitz Social Anxiety Scale – Self Report (LSAS-SR)

The LSAS-SR was the primary outcome measure. The LSAS-SR measures fear and avoidance related to social situations using 24 items (Fresco et al., 2001). Respondents are asked to rate their fear and anxiety regarding a social situation on a scale between 0 (no fear or anxiety) and 3 (severe fear or anxiety). They also rate how often they avoid the situation or scenario, ranging from 0 (never) to 3 (usually). The ratings are summed up to provide a general rating of social anxiety, ranging between 0 and 144. The self-report version of the scale has been noted to have excellent internal consistency (Cronbach’s α = 0.95) and a 12-week test–retest reliability of *r* = .83 (Baker et al., 2002). It has been validated for internet administration (Hedman et al., 2010). The questionnaire was administered online as a screening tool before the treatment began, weekly during the treatment (at a fixed day each week which was also the same time as participants were sent a new module if they had completed the previous module), and at post treatment.

#### Secondary Outcome Measures

All secondary outcome measures were administered at the screening (before this part of the study took place), at the pretreatment time point (the start of the current study), and at the post treatment time point.

##### Social Interaction Anxiety Scale (SIAS)

The SIAS consists of 20 items aimed at measuring the respondent’s anxiety during social interactions (Heimberg et al., 1992). Ratings are made on a Likert scale from 0 (not at all characteristic or true of me) to 4 (extremely characteristic or true of me), with the total sum ranging from 0 to 80. Psychometric properties include excellent internal consistency (Cronbach’s α = .93) and a 12-week test–retest reliability of *r* = .92 (Mattick & Clarke, 1998).

##### Social Phobia Scale (SPS)

The SPS consists of 20 items administered with the intention of measuring respondents’ fear of evaluation in social situations (Heimberg et al., 1992). Ratings are made on a Likert scale from 0 (not at all characteristic or true of me) to 4 (extremely characteristic or true of me), with a total sum range of 0 to 80. Psychometric properties include an internal consistency (Cronbach’s α = .94) and a 12-week test–retest reliability of *r* = .93 (Mattick & Clarke, 1998).

##### Beck Anxiety Inventory (BAI)

The BAI consists of 21 items that measure the physiological and cognitive symptoms of anxiety (Beck et al., 1988). Ratings are made on a four-point Likert scale, with possible sum scores ranging from 0 to 63. The instrument’s psychometric properties include internal consistency (Cronbach’s α = .92) and a one-week test–retest reliability of *r* = .75 (Beck et al., 1988).

##### Montgomery Åsberg Depression Rating Scale – Self Report (MADRS-S)

The MADRS-S is a nine-item scale measuring symptoms of depression based on the 10-item clinician-administered version of the scale (Montgomery & Åsberg, 1979). Respondents rate the frequency of cognitive, emotional, and physiological symptoms during the past three days on a seven-point scale. Total sum scores can range from 0 to 54, with higher scores indicating an increased severity of symptoms. Psychometric properties for the self-report version have been reported to include an internal consistency (Cronbach’s α = .84) and a one-week intraclass correlation of .78 (Fantino & Moore, 2009). Both BAI and MADRS-S are validated for internet use (Thorndike et al., 2009).

##### Quality of Life Inventory (QoLI)

The QoLI is a 16-item instrument measuring respondents’ subjectively rated quality of life (Frisch et al., 1992). The respondent is asked to indicate how important a specific domain is on a scale from 0 (not important) to 2 (very important), and then how satisfied they are with their current situation within that domain. The two ratings are multiplied and divided by the number of areas that the respondent considers to be somewhat or very important. The test–retest coefficient was measured between *r* = .80 and .91 (mean duration between measurements = 33 days) during the validation of the instrument. The range of values for internal consistency was reported as being between Cronbach’s α = .77 and .89. The QoLI, has been validated for internet use (Lindner et al., 2013).

### Power

A formal power analysis was not conducted, as this was a spin-off study following the first phase of the trial. However, given the sample size and a power of 80% and *p* < .05, we had statistical power to detect an effect size of *d* = 0.40 on the LSAS-SR. This would correspond to a clinically relevant effect, with the expected direction being the superiority of scheduled support over support on demand.

### Statistical Analyses

Statistical analyses were conducted using R version 4.0.3 (R Core Team, 2020) and SPSS version 25. Across the analyses, the alpha level was set to .05. Confidence intervals were reported at 95%. The assumption of normality was controlled using Shapiro-Wilks tests. Tests of pretreatment differences and differences between responders and non-responders on the post treatment assessment were evaluated using independent sample *t*-tests, Mann-Whitney *U* tests (when the assumption of parametric data was not met) and Fisher’s exact tests. Independent *t*-tests were also used to investigate potential differences in the number of modules accessed (i.e., read) and completed (defined as completing the exercises in a module). A multiple regression model using residualized change scores as the dependent variable was used to investigate the relationship between completion of modules and change in the primary outcome measure. The data were analyzed according to the intention-to-treat principle (ITT), meaning that all available data were included in the analysis and all randomized participants were included in the analysis. The post treatment data from one of the participants in the scheduled support condition was flagged, as the scores on all the outcome measures were 0 (including both symptom measures, such as the LSAS and the quality-of-life ratings). Due to this likely mistake/error the post treatment data for this participant were marked as missing.

The model used to investigate the outcome of the primary outcome measure (LSAS-SR) was a mixed-effects model fitted using the *lme4* package (Bates et al., 2015). Model fit, including the form of change and covariance structure for the primary outcome where we had weekly measurements, was investigated iteratively using a likelihood ratio test (by using the *ANOVA* function in R). The final model for the primary outcome measure incorporated a linear rate of change, random intercept and slope, and an unstructured residual variance structure. For the secondary outcomes with only two data points, we estimated a random intercept but not a random slope. Q-Q plots were used to assess the normal distribution of the residuals for all the mixed models. Significance for the fixed effects in the models was evaluated using the Wald test, in which the estimate was divided by the standard error and compared against a *z*-distribution. Inferences about the random effects of the model for the primary outcome measure are not evaluated by the Wald test but rather from the estimated confidence intervals, where an interval not containing zero is interpreted in the same way as a significant *p*-value. Confidence intervals were calculated using the *ConfintMermod* function with the profile method. The models were estimated using restricted maximum likelihood estimation, thus making use of all available data. The use of maximum likelihood estimation is one of two recommended approaches for dealing with missing data (Schafer & Graham, 2002). Maximum likelihood estimators provide unbiased estimates in situations where data can be assumed to be missing at random (MAR), meaning that the data are not missing systematically as a function of the would-be value. This is a less restrictive assumption than missing completely at random (MCAR), where missingness is assumed to be independent of both the would-be value and the values of the other variables.

Due to differences in means between the conditions at pretreatment for the outcome measures, the parameter deemed to be of interest was the time x group interaction rather than the endpoint difference between the conditions. The conditions were coded as scheduled support = -0.5 and support on demand = 0.5.

The Cohen’s *d* between-group effect size for the estimated parameters of the models was calculated with the *lme.dscore* function using the Satterwaites degrees of freedom according to the formula *d* = 2*t*/Sqrt(df) (Rosenthal, 1994). Observed within-group effect sizes were calculated with the pooled standard deviations from the pre- and post treatment measurements. Between-group effect sizes were interpreted according to the recommendation provided by Cohen, with 0.20, 0.50, and 0.80 corresponding to small, moderate, and large effect sizes, respectively (Cohen, 1988).

Reliable change/deterioration was calculated according to the formula provided by Jacobson and Truax (1991), where the pretreatment mean was subtracted from the post treatment mean and divided by the pooled standard deviation adjusted for the instrument’s test–retest reliability (Jacobson & Truax, 1991). The critical value for the LSAS-SR was set at ± 28 points.

## Results

### Baseline Characteristics

The demographic characteristics of the sample are presented in Table 2. The conditions did not differ significantly with regard to age, gender, civil status, or education level, all of which were *p* > .05.

**Table 2:** Demographic and Clinical Characteristics of the Sample (n = 99)

Characteristic	Scheduled support		Support on demand			
	*M*	*SD*	*M*	*SD*	*t*(97)	*p*
**Age**	39.44	10.60	37.59	11.42	0.84	.33
	*n*	%	*n*	%	χ^2^	*p*
Gender						
Female	17	34.0	22	44.9	1.23	.32
Male	33	66.2	27	55.1		
Civil status						
Single	19	38.0	17	32.7	0.23	.63
In a relationship/Married	31	62.0	32	65.3		
Highest educational degree						
Primary school	1	2	3	6.1	5.98	.11
High school	12	24	13	26.5		
University	31	62	20	40.8		
Other post-secondary education	6	12	13	26.5		
	*M*	*SD*	*M*	*SD*	*t*(97)	*p*
Outcome measure						
LSAS	58.76	24.14	69.71	21.99	-2.36	.020
SIAS	43.24	15.09	48.82	14.14	-2.74	.007
QoLI	1.29	1.66	0.22	1.59	3.30	.001
	*M*	*SD*	*M*	*SD*	*U*	*p*
SPS	28.80	13.73	36.69	14.94	2.822	.005
BAI	11.56	7.30	16.82	8.45	3.211	.001
MADRS-S	12.50	6.12	17.02	7.63	3.007	.003

*Note.* LSAS = Liebowitz Social Anxiety Scale – Self-rated; SIAS = Social Interaction Anxiety Scale; SPS = Social Phobia Scale; BAI = Beck Anxiety Inventory; MADRS-S = Montgomery-Åsberg Depression Rating Scale – Self-rated; QoLI = Quality of Life Inventory; *U* = Mann-Whitney *U*-test statistic.

### Attrition, Missing Data, Activity Statistics, and Adherence

Five participants in the support-on-demand group (10%) dropped out of the study during the treatment period. One of the participants from the scheduled support group dropped out during the treatment period (2%). There was no significant difference in the proportion of dropouts between the two conditions, χ^2^(1) = 2.93, *p* = .087. For the sample as a whole, data were provided for 87% of the primary outcome measurements during the study. A total of 49 participants (98%) in the group with scheduled support completed all post treatment measurements. In the support on demand group, 42 participants (86%) completed all post treatment measures. The groups differed significantly in this regard, χ^2^(1) = 5.03, *p* = .025, suggesting that the support on demand group was less likely to complete the post treatment measurement. For the clinical and demographic variables, there were no significant differences between those who completed the post treatment assessment and those who did not (all *p* > .05).

Activity statistics are presented in Table 3. For the support on demand group, the average total number of messages sent to the clinician during the treatment was 0.6 (*SD* = 1.10, range = 0–4), which was much lower than in the scheduled support group (*M* = 15.04, *SD* = 8.03, range = 0–45) (*p* < .001). On average, the support on demand group accessed 77.4% of the modules, while the scheduled support group accessed 83.3%. This difference was not significant, *p =* .359. However, participants with scheduled support completed significantly more modules (79.2% on average) than participants in the support on demand group (64.2% on average), *p* < .001. The group receiving scheduled support also posted more on the discussion forum (*p* < .001). As expected, clinicians supporting the scheduled support group also spent more time on average attending to their participants than the clinician responsible for the support on demand group (*p* < .001).

**Table 3:** Statistics on Activity and Comparisons Between the Conditions

Variable	Scheduled support	Support on demand	*t*(97)	*p*
	*M* (*SD*)	*M* (*SD*)		
Number of emails sent by participants to the clinician	15.04 (8.03)	0.44 (1.09)	12.48	< .001
Number of posts made on the discussion forum	11.76 (7.99)	3.57 (5.24)	6.01	< .001
Modules accessed during treatment (out of nine)	7.50 (2.49)	6.97 (2.82)	0.92	.359
Modules completed during treatment (out of nine)	7.13 (2.48)	5.78 (2.89)	2.36	.020
Clinician time per week and participant (minutes)	14.00 (6.08)	0.6 (1.10)	15.30	< .001

The multiple regression model showed no significant predictive value in residualized gain score for neither condition, β = -.33, *p* = .226, or the number of completed modules, β *=*.08, *p* = .585. There was, however, an interaction between condition and module completion for the gain scores, β *=*.55, *p* = .045. This suggests that the number of completed modules was significantly related to a greater reduction in symptoms but only in the support on demand group. The explained variance in the LSAS-SR outcome was *R*^2^ = 0.141.

### Primary Outcome

#### Liebowitz Social Anxiety Scale – Self Report (LSAS-SR)

Observed means including effect sizes are reported in Table 4. For the LSAS-SR ratings, the mixed-effects model revealed significant heterogeneity in both the intercept, *SD* = 23.26, 95% CI [19.99, 26.81], and the slope, *SD* = 2.43, 95% CI [2.05, 2.84], across the sample. Additionally, the results showed a strong correlation between intercept and slope, *r* = -.53, 95% CI [-.67, -.35], suggesting that higher initial ratings were related to a steeper decline in symptoms during the treatment period. The fixed effects showed a significant difference between the groups at pretreatment, *b* = 11.40, 95% CI [2.02, 20.79], *SE* = 4.79, *p* = .019, indicating that the support on demand group had significantly higher ratings on the LSAS-SR at the start of the treatment. There was a significant linear decrease in symptoms over each unit of time (one week) for the entire sample, *b* = -2.61, 95% CI [-3.13, -2.09], *SE* = 0.26, *p* < .001. The interaction between time and group was not significant, *b* = -0.07, 95% CI [-1.10, 0.97], *SE* = 0.53, *p* = .898, suggesting that there was no significant difference in slope between the two conditions. The effect size for this comparison was *d* = 0.02, 95% CI [-0.37, 0.43], with the slight difference favoring the condition with support on demand.

**Table 4:** Observed Means for the Outcome Measures at Pre- and Post-Treatment With Within-Group Effect Sizes

Outcome measure	Pre-treatment		Post-treatment		Observed within-group effect size
	*M* (*SD*)	*n*	*M* (*SD*)	*n*	*d* [95% CI]
LSAS					
Scheduled	58.76 (24.14)	50	37.80 (22.79)	49	-0.89 [-1.31, -0.48]
On demand	69.71 (21.99)	49	47.62 (22.43)	42	-1.00 [-1.43, -0.56]
SIAS					
Scheduled	43.24 (15.09)	50	32.90 (16.53)	49	-0.65 [-1.06, -0.25]
On demand	48.82 (14.14)	49	38.43 (15.96)	42	-0.69 [-1.12, -0.27]
SPS					
Scheduled	28.80 (13.73)	50	18.71 (13.27)	49	-0.75 [-1.16, -0.34]
On demand	36.69 (14.94)	49	23.76 (15.11)	42	-0.86 [-1.29, -0.43]
BAI					
Scheduled	11.56 (7.30)	50	8.18 (7.62)	49	-0.45 [-0.85, -0.05]
On demand	16.82 (8.45)	49	11.05 (7.68)	42	-0.71 [-1.14, -0.29]
MADRS-S					
Scheduled	12.50 (6.12)	50	7.86 (6.20)	49	-0.75 [-1.16, -0.35]
On demand	17.02 (7.63)	49	10.76 (7.36)	42	-0.83 [-1.26, -0.40]
QoLI					
Scheduled	1.29 (1.66)	50	2.04 (1.67)	49	0.45 [0.05, 0.85]
On demand	0.22 (1.59)	49	0.88 (1.61)	42	0.41 [0.00, 0.82]

*Note.* LSAS = Liebowitz Social Anxiety Scale – Self-rated; SIAS = Social Interaction Anxiety Scale; SPS = Social Phobia Scale; BAI = Beck Anxiety Inventory; MADRS-S = Montgomery-Åsberg Depression Rating Scale – Self-rated; QoLI = Quality of Life Inventory.

### Secondary Outcomes

#### Social Interaction Anxiety Scale

The model did not indicate a significant initial difference between the conditions in the SIAS ratings *b* = 5.08, 95% CI [-4.46, 14.62], *SE* = 4.89, *p =* .301. Overall, the SIAS scores decreased during the treatment, *b* = -10.12, 95% CI [-16.42, -4.26], *SE* = 1.36, *p* < .001. The interaction between time and group was not significant, *b =* 0.50, 95% CI [-4.46, 5.81], *SE* = 2.71, *p =* .855. This difference in change equaled an effect size of *d* = 0.17, 95% CI [-0.35, 0.45] in favor of the group with scheduled support.

#### Social Phobia Scale

The analysis showed a significant initial difference between the conditions on the SPS, *b* = 10.04, 95% CI [1.32, 18.75], *SE* = 4.47, *p* = .026. There was a significant overall average decrease from pre to post treatment, *b* = -11.03, 95% CI [-15.88, -4.29], *SE* = 1.21, *p* < .001. The time x group interaction was not statistically significant, *b* = -2.15, 95% CI [-6.87, 2.59], *SE* = 2.42, *p* = .377. This difference in change corresponded to an effect size of *d* = 0.18, 95% CI [-0.22, 0.59] in favor of the support on demand group.

#### Beck Anxiety Inventory

The groups differed significantly in their initial BAI ratings, *b* = 7.49, 95% CI [2.61, 12.38], *SE* = 2.50, *p* = .003. There was an overall decrease in the BAI scores, *b* = -4.36, 95% CI [-5.74, -2.99], *SE* = 0.70, *p* < .001. The interaction between time and group was not statistically significant, *b* = -2.24, 95% CI [-6.83, 2.04], *SE* = 1.40, *p* = .115. The corresponded to an effect size of *d* = 0.34, 95% CI [-0.31, 1.03] favoring the support on demand group.

#### Montgomery Åsberg Depression Rating Scale – Self Report

There was a significant pretreatment difference in the MADRS-S scores, *b* = 5.73, 95% CI [1.40, 10.07], *SE* = 2.22, *p* = .011. After the treatment period, the analysis showed a significant decrease for the sample, *b = -*5.30, 95% CI [-6.54, -4.08], *SE* = 0.63, *p* < .001. The interaction between time and group was again not significant, *b* = -1.21, 95% CI [-3.68, 1.24], *SE* = 1.26, *p* = .338. The effect size for the difference in change between the groups was *d* = 0.20, 95% CI [-0.21, 0.61] favoring the support on demand group.

#### Quality of Life Inventory

The groups did not differ significantly in their pretreatment QoLI scores, *b* = -0.88, 95% CI [-2.46, 0.46], *SE* = 0.47, *p* = .064. The sample showed a significant increase in the QoLI during the treatment period, *b* = 0.66, 95% CI [0.23, 1.17], *SE* = 0.12, *p* < .001. The groups did not differ significantly in their changes during this period, as indicated by the interaction between group and time, *b* = -0.20, 95% CI [-1.01, 0.86], *SE* = 0.24, *p* = .423. The effect size for the difference in change between the groups during the treatment was *d* = 0.17, 95% CI [-0.76, 0.89] favoring the group with scheduled support.

### Reliable Change/Deterioration

In total, 27 of the respondents (27%) at post treatment met the criteria for reliably improving during the treatment period. None of the participants were classified as reliably deteriorated. The proportion of clinically significantly improved participants did not differ between the scheduled support group (*n* = 12) and the support on demand group (*n* = 15), Fisher’s exact *p* = .504.

## Discussion

The aim of this study was to investigate the effects of a support on demand model for delivering ICBT targeting SAD relative to a standardized form of clinical support. Results suggested overall significant reductions in symptoms of SAD and related psychopathology, along with an increase in quality of life with no significant between-group differences. The effect sizes for the estimated within-group pre-to-post comparisons on the measures of social anxiety were all large. The reduction in the symptoms of social anxiety is consistent with earlier findings indicating that ICBT can be an effective alternative for treating SAD (Guo et al., 2021). Results further suggest that active therapist guidance may be reduced with support-on-demand without significant loss of treatment gains.

The lack of significant differences in change between the groups and the nonexistent-to-small between-group effect sizes are in line with the notion that support on demand can be a way of delivering ICBT for SAD. The findings also add to the literature on comparisons between the support on demand format and traditional ways of administering ICBT with weekly clinician support. Like earlier studies (Hadjistavropoulos et al., 2017; Hadjistavropoulos et al., 2019), the analyses indicated that the two conditions did not differ significantly in change during the treatment. Although the randomization “failed” as the groups differed at baseline, the lack of significant interactions between time and group suggests that support on demand can be a sufficiently effective way of delivering ICBT compared to the more established clinician-guided format. That has positive implications for scalability (Andersson et al., 2019). As expected, participants in the support on demand condition required significantly less clinician time per module than scheduled support participants. The average number of requests for help and/or feedback was low (*M* = 0.44), and none of the participants sent more than four messages to the clinician. Extrapolating from this, it is likely that a support on demand model could be a resource-effective way of disseminating ICBT for SAD, given that there are clinicians who are prepared to provide support when needed. This differentiates on demand ICBT from fully self-guided versions in which contact with clinicians is not offered or only possible in urgent cases. However, the reduced need for clinician support gives credibility to the idea that ICBT could be administered to a larger number of patients with relatively few clinicians, thus making it easier to disseminate in contexts where a lack of trained clinicians is a problem. As unguided interventions have sometimes been deemed less effective than interventions with scheduled support (Ciuca et al., 2018), a support on demand model could serve as a compromise, making it possible to disseminate more broadly with the decreased need for clinician support.

While the two guidance conditions produced comparable reductions in social anxiety, there were some differences in the activity levels between them. Participants in the group with scheduled support completed more modules, sent more emails to their clinician, and made more posts on the discussion forum than participants with support on demand. It is unclear whether activity levels such as these are important in relation to the outcome of ICBT in general, but the fact that module completion predicted a stronger reduction in symptoms in the support on demand group could be important. Future studies could investigate this relationship and whether module engagement in the support on demand condition can be increased with the addition of optional components such as personalized reminders (Hilvert-Bruce et al., 2012). For unguided ICBT, treatment credibility has also been noted as relevant to adherence (Nordgreen et al., 2012), and this would be interesting to investigate in relation to the on-demand format. Of note is that a significantly larger proportion of the participants in support on demand failed to complete the post treatment measures. This is likely due to a larger dropout rate during the treatment period. As the participants who provided post treatment ratings did have lower pretreatment scores on the BAI, the results for this outcome measure should be interpreted with caution. Inquiring about the reasons for dropout and non-adherence could be important going forward. Such information may inform decisions about who the support on demand format is a good match compared to a scheduled and structured mode of clinician support.

The results of the study should be viewed with some limitations in mind in addition to the fact that it is a file drawer study and hence could be less relevant even if technology in many ways has remained the same. First, the sample size was suboptimal for testing the differences between the two active treatment conditions. As Cuijpers et al. (2019) noted, studies investigating the components of psychological treatments often have far too small a sample to serve as outright non-inferiority trials (Cuijpers et al., 2019). It is important to note that the present study was not intended as such but rather a proof-of-concept trial regarding the ability to provide a new way of guiding participants through an ICBT treatment. When the SOFIE-6 study was conducted, no such trials had been published apart from studies testing the added value of scheduled telephone calls (Andersson et al., 2003; Kenwright et al., 2005). The results should not be interpreted as conclusive but rather as an indication that support on demand can be feasible in the treatment of SAD and possibly other conditions. Additional, better-powered trials are needed, along with studies on change mechanisms, as we do not know what works for whom in terms of support.

Second, the randomization procedure did yield unbalanced group in terms of their pre-treatment differences. Though the statistical analyses focused on the differences in change over time, rather than just the endpoint differences between the conditions, this should be kept in mind when interpreting the results and the outcome ratings.

Third, the study lacked data on some variables that might be of interest in addition to ratings of symptoms. For example, we did not measure treatment satisfaction or working alliance during and at post treatment. Although the groups did not differ significantly with regard to changes in the outcome measures, such information could be valuable when seeking to understand other factors that might be important, such as adherence and module completion.

Fourth, data were not collected beyond the post treatment assessment. Although the comparison of changes between the two conditions did not differ during the treatment period, the findings by Ivanova et al. (2016) indicated that differences in effect may occur later (Ivanova et al., 2016). While the long-term effects of ICBT in general is favorable (Andersson, 2018), future studies should strive to investigate the long-term effects of different support forms.

Lastly, though both the conditions had access to a forum, the condition with scheduled support made use of this function significantly more often. Given that a similar forum may produce symptom reductions (Griffiths et al., 2009), the fact that the design of the present study did not control for the specific effect of forum usage is a limitation.

In conclusion, the present study provides support for the role of support on demand as a way of delivering ICBT, and that the format is suitable in the treatment of SAD. It can also serve as an example of the importance of still reporting studies in which the data (in this case, randomization group differences) do not fulfill expectations. The findings are important, as groups exhibited very similar symptom trajectories during the treatment period, regardless of whether they received scheduled weekly support, or had the option to contact a clinician when needed. Additionally, no significant differences were found for any of the secondary measures. Given the small number of studies testing the support on demand format, we look forward to replications and systematic reviews when a sufficient number of trials have been conducted.
